# An efficient multi-stage fermentation strategy for the production of microbial oil rich in arachidonic acid in *Mortierella alpina*

**DOI:** 10.1186/s40643-017-0138-8

**Published:** 2017-01-18

**Authors:** Wen-Jia Wu, Ai-Hui Zhang, Chao Peng, Lu-Jing Ren, Ping Song, Ya-Dong Yu, He Huang, Xiao-Jun Ji

**Affiliations:** 1 College of Biotechnology and Pharmaceutical Engineering, Nanjing Tech University, No. 30 South Puzhu Road, Nanjing, 211816 People’s Republic of China; 2 School of Pharmaceutical Sciences, Nanjing Tech University, No. 30 South Puzhu Road, Nanjing, 211816 People’s Republic of China; 3 State Key Laboratory of Materials-Oriented Chemical Engineering, Nanjing Tech University, No. 5 Xinmofan Road, Nanjing, 210009 People’s Republic of China; 4 Jiangsu National Synergetic Innovation Center for Advanced Materials (SICAM), No. 5 Xinmofan Road, Nanjing, 210009 People’s Republic of China; 5 Beijing Key Laboratory of Nutrition Health and Food Safety, COFCO Nutrition and Health Research Institute, Beijing, 102209 People’s Republic of China

**Keywords:** Arachidonic acid, *Mortierella alpina*, Multi-stage fermentation, Aeration, Agitation, Morphology

## Abstract

**Background:**

Fungal morphology and aeration play a significant role in the growth process of *Mortierella alpina*. The production of microbial oil rich in arachidonic acid (ARA) in *M. alpina* was enhanced by using a multi-stage fermentation strategy which combined fed-batch culture with precise control of aeration and agitation rates at proper times.

**Results:**

The fermentation period was divided into four stages according to the cultivation characteristics of *M. alpina*. The dissolved oxygen concentration was well suited for ARA biosynthesis. Moreover, the ultimate dry cell weight (DCW), lipid, and ARA yields obtained using this strategy reached 41.4, 22.2, 13.5 g/L, respectively. The respective values represent 14.8, 25.8, and 7.8% improvements over traditional fed-batch fermentation processes.

**Conclusions:**

This strategy provides promising control insights for the mass production of ARA-rich oil on an industrial scale. Pellet-like fungal morphology was transformed into rice-shaped particles which were beneficial for oxygen transfer and thus highly suitable for biomass accumulation.

## Background

Arachidonic acid, (5, 8, 11, 14-cis-eicosatetraenoic acid, ARA), a representative of the omega-6 group of essential polyunsaturated fatty acids (PUFAs), acts as a precursor for eicosanoid hormones such as prostaglandins, leukotrienes, and thromboxanes (Ji et al. 2014a). The application of ARA as the active ingredient in drugs and food additives thus has great potential. Owing to its unique physiological functions, it has been widely applied in the food industry as well as cosmetics, medicine, and many other fields (Ward and Singh 2005). For a long time, egg yolk, animal liver, and adrenal glands were the main sources of ARA. However, their low intrinsic ARA content (Higashiyama et al. 2002) restricts their application, and it is not possible to source sufficient material for use of ARA in infant formula. On the other hand, ARA-rich oil derived from the oleaginous fungus *Mortierella alpina* has received GRAS status from the US FDA in 2001 (Ryan et al. 2010), and *M. alpina* is regarded as one of the most promising candidates for the mass production of ARA-rich oil (Ji et al. 2014a). The ARA biosynthesis pathway in *M. alpina* proceeds via the formation of C16 or C18 saturated fatty acids, which are further modified through a series of elongation and desaturation steps, culminating in the formation of ARA. It is known that these reactions require NADPH, an electron transport system, a terminal desaturase, and molecular oxygen (Ward and Singh 2005).

In general, fungal mycelia are brittle and physically weak. Therefore, the agitation rate in mechanically stirred bioreactors, which are normally used for the production of ARA-rich oil, has to be controlled within a very precise range. High agitation rates increase the shear forces, which can break mycelial integrity and influence the broth characteristics. Low agitation rates, on the other hand, lead to a low dissolved oxygen concentration insufficient for ARA biosynthesis. Overall, mycelial morphology has a strong effect on the physical properties of the broth and often leads to a number of different problems in large bioreactors with respect to gas dispersion, as well as mass and heat transfer (Higashiyama et al. 2002). There are many reports that discuss the size and shape of fungal mycelial pellets (Xu et al. 2010; Tai et al. 2010), but little is known about the true features of the internal pellet structure, including geometry and mycelial viability (Hamanaka et al. 2001). Interestingly, pellets with a moderate compactness are the more productive morphological form for the production of ARA-rich oil, compared to free filamentous mycelia. Therefore, controlling proper aeration and agitation rates in the whole process to balance the contradiction between these two factors is vitally important for the fermentation of fungal producers of ARA-rich oils. There have been some attempts to fulfill this objective by controlling the aeration (Higashiyama et al. 1999; Nie et al. 2014) and agitation rates (Higashiyama et al. 1999; Peng et al. 2010), respectively. ARA yields in these reports reached 4.7 g/L by strictly monitoring the mycelial morphology and employing a two-stage control strategy for the aeration rate, which represents an increase of 38.2% (Gao et al. 2016). However, until now, no efforts have been made to simultaneously evaluate the aeration and agitation rate in relation to the proper mycelial morphology for increasing the biomass yield of the filamentous fungus *M. alpina*.

In this study, an innovative multi-stage strategy was investigated to optimize ARA productivity in bioreactors. We thereby aimed at balancing the contradiction between the aeration and agitation controls required for optimal dissolved oxygen concentration and fungal morphology, respectively. The strategy was further assessed regarding its effectiveness in improving the biomass yield, which reached >40 g/L. This approach gives a detailed insight into the mycelial morphology control of oil-producing filamentous fungi and will provide guidance for the large-scale production of ARA and similar polyunsaturated fatty acids.

## Methods

### Microorganism

*Mortierella alpina* R807 (CCTCC M 2012118), preserved in the China Center for Type Culture Collection, was used in the present study. It was maintained on potato dextrose agar (PDA) slants by culturing for 10 days at 25 °C, and transferred every 3 months.

### Culture medium

Slant medium: Potato dextrose agar (PDA). The PDA medium contained (g/L): potatoes 200; glucose 25; agar 20. Inoculation medium (g/L): yeast extract (Angel Yeast Co., Ltd, China) 6; glucose 30; KH_2_PO_4_ 3; NaNO_3_ 3; MgSO_4_·7H_2_O 3. Fermentation medium (g/L): yeast extract 10; glucose 80; KH_2_PO_4_ 4; NaNO_3_ 3; MgSO_4_·7H_2_O 0.6; initial pH 6.0.

### Fermentation methods

Pellets of *M. alpina* were used to inoculate the PDA slants which were cultivated at 25 °C. After 12–15 days of incubation, a loop was used to transfer mycelial material into deep 250 mL baffled flasks containing 50 mL inoculation medium, and the cultures were subsequently incubated for 2 days at 25 °C under constant orbital shaking at 125 rpm. Fed-batch fermentations were carried out in a 7.5 L bioreactor (New Brunswick Scientific, USA) containing 5 L of fermentation medium.

The multi-stage process was carried out according to our proposed stepwise aeration and agitation control strategy. The aeration rate was set at 6 L/min to achieve an aeration rate of 1.2 volumes of air per volume of liquid per minute (vvm), without agitation in stage I (0–48 h). The agitation rate was increased stepwise from 50 to 150 revolutions per minute (rpm) in stage II and subsequently kept constant at 200 rpm until the end of the fermentation. The aeration rate was set at 1.0 vvm from step II to step IV. Glucose (500 g/L stock solution) was fed into the fermentation broth during the entire fermentation process to maintain the glucose concentration at 5–20 g/L. Samples comprising 100 mL of the fermentation broth were taken periodically for further examination.

### Determination of dry cell weight (DCW) and glucose concentration

Aliquots comprising 100 mL of the fermentation broth were used to determine the DCW using the filtration method. The broth samples were transferred to a suction filter under 0.1 MPa negative pressure. The cell pellet was washed twice with distilled water and dried at 60 °C to constant weight (12 h). An aliquot comprising 1 mL of fermentation broth was transferred to a centrifuge tube, centrifuged at 3000×*g* for 3 min, and the resulting supernatant was used to measure the glucose concentration, which was determined enzymatically using a simultaneous Bioanalyzer (SBA-40C, Institute of Biology, Shandong Academy of Sciences, China).

### Total lipids (TLs)

The dry cell material was ground into a fine powder for lipid extraction and fatty acid determination. A 2 g aliquot of the resulting powder was loaded onto a Soxhlet extractor with 150 mL chloroform/methanol (2:1, v/v) and extracted for 8 h at 75 °C. Finally, the solvent was removed on a rotary evaporator and recycled, with TLs remaining as evaporation residue (Ji et al. 2014b).

Fatty acid methyl esters (FAMEs) were prepared according to the established method (Ji et al. 2014b; Ren et al. 2009) as follows: 1.5 mL n-hexane and 0.2 mL 0.5 M KOH– methanol were added to a centrifuge tube containing 0.1 g of the powdered dry cells, and mixed thoroughly by vortexing for 3 min, followed by stewing for 15 min. A 0.3 mL aliquot of the resulting upper phase was combined with 0.5 mL distilled water in another centrifuge tube and the tube was centrifuged at 5000×*g* for 3 min. The upper phase containing FAMEs was applied to a Thermo Finnigan trace GC2000 DSQ gas chromatograph equipped with a 30 m × 0.25 mm × 0.25 µm DB-23MS capillary column (Agilent Technologies). The column temperature was increased from 80 to 200 °C at 40 °C/min, and subsequently to 300 °C at 10 °C/min. The temperature of both the injector and detector was set to 250 °C. Nitrogen was used as the carrier gas at 1 mL/min. Peaks were identified using authentic standards of the corresponding fatty acid methyl esters (Sigma-Aldrich). Fatty acids were quantified based on their corresponding peak areas relative to the peak areas of the standards.

## Results and discussion

### Fermentation disparities between batch and fed-batch protocols

**Fig. 1:**
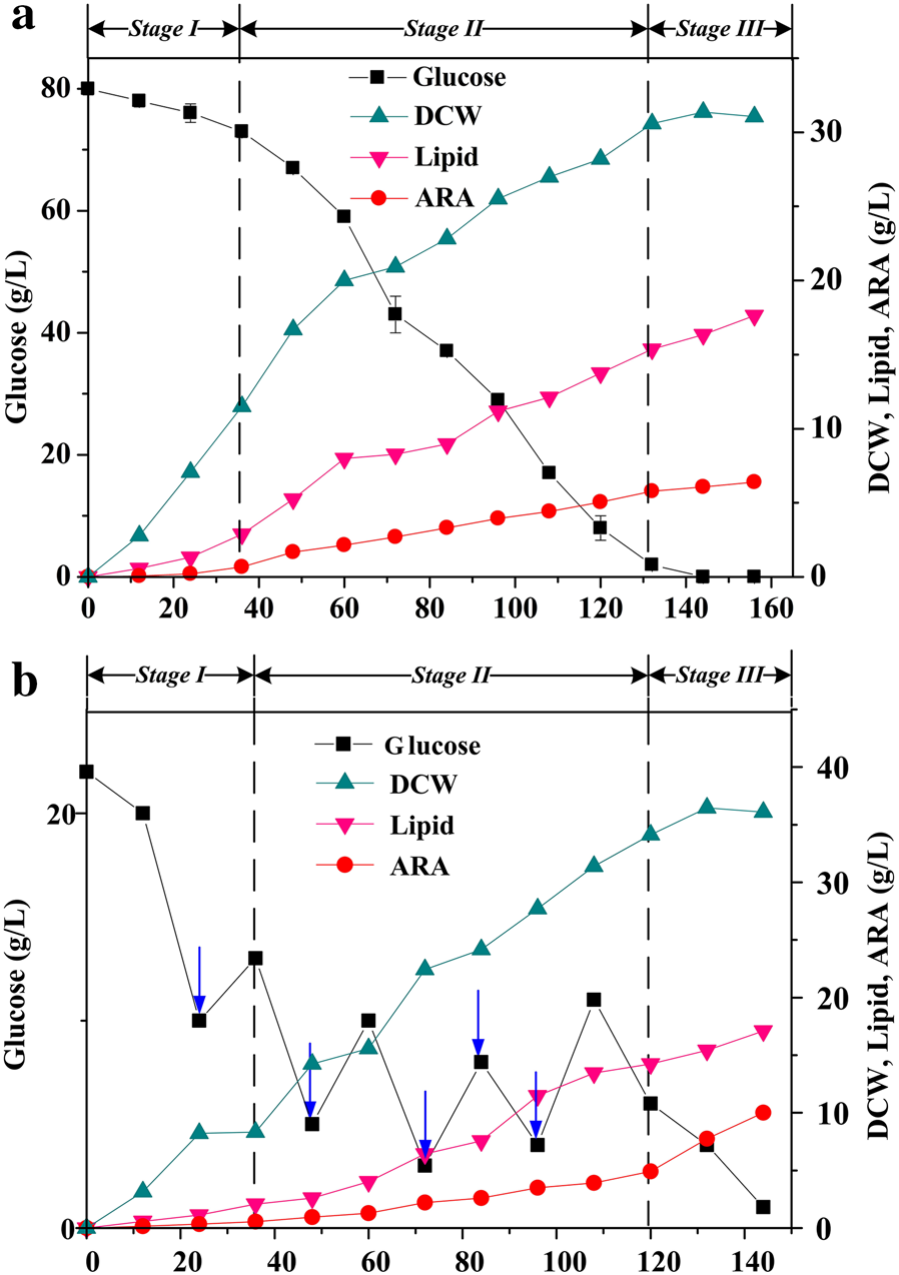
Time-course of different fermentation parameters from fermentations using *M. alpina* for the production of ARA-rich oil; **a** batch; **b** fed-batch

The effects of batch and fed-batch fermentation on DCW, lipid, ARA contents, and ARA production were investigated using a 7.5 L bioreactor (Fig. 1). Though batch fermentation was found to be optimal for growth and total lipid production, ARA was synthesized more rapidly in fed-batch cultures. The lipid concentration reached a maximum value of 17.6 g/L at 6.5 days. However, higher ARA contents (42.8%) and ARA yield (10.0 g/L) were obtained with fed-batch fermentation. These results suggest that the optimal culture conditions for lipid accumulation and ARA biosynthesis are different. A higher C/N ratio was achieved in batch fermentations, which stimulated lipid accumulation. However, it also led to lower ARA biosynthesis. Furthermore, the C/N ratio is an important fermentation parameter which can affect mycelial morphology (Koike et al. 2001; Park et al. 2001), and it has been demonstrated previously that the morphology of *M. alpina* mycelia has a strong effect on physical properties of the broth, which in turn might lead to poor mass transfer performance. Due to the difficulty of controlling the mycelial morphology of *M. alpina* under constant aeration and agitation rates, the dry cell weight obtained in fed-batch fermentation (36.1 g/L) was not much higher than what was obtained in batch fermentation (31.1 g/L). However, the ARA contents and ARA yield (42.8% and 10.0 g/L, respectively) were clearly enhanced over the batch fermentation. A low initial glucose concentration is usually used to shorten the lag phase of fungal growth (Zhu et al. 2006). The glucose consumption during this stage was rapid, and the glucose was used up almost completely by day 6. The ARA productivity achieved by this method was 1.67 g/L day^−1^, which is 1.70-fold higher than what was obtained in batch fermentations.

### Controlling the morphology of *Mortierella alpina* using a multi-stage fermentation strategy

**Fig. 2:**
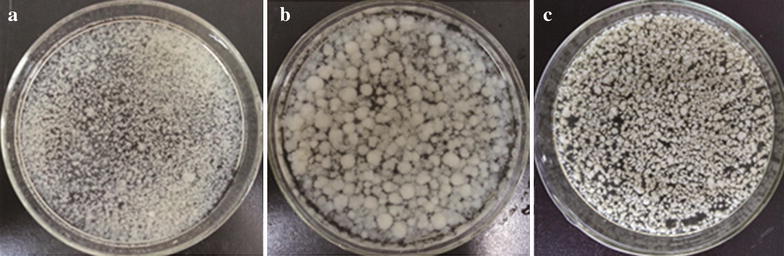
Morphology of *M. alpina* mycelia cultured using different fermentation strategies. **a** At the end of the fed-batch fermentation; **b** at 48 h of the multi-stage strategy; **c** at the end of the multi-stage fermentation strategy

Seed culture morphology was found to be a significant factor in fermentations producing ARA-rich oil. This was directly due to the effects of mycelial morphology on the physical properties of the fermentation broth (Higashiyama et al. 2002). Thus, mycelial morphology is considered to be a key parameter in ARA fermentation, and the fungus must consequently be cultivated in the correct morphological form in order to obtain maximal ARA concentration (Ji et al. 2014b). Although feather-like hyphal filaments (Fig. 2a) were observed to be optimal for ARA production at low densities (Park et al. 1999), this morphology is disadvantageous at high cell densities because viscosity of the ferrmentation broth may be increased to an extent that oxygen transmission becomes limited (Wynn and Ratledge 2005).

In stage I (from 0 to 48 h), pellet-like mycelia of *M. alpina* were formed using baffled shake flasks and transferred into the nutrient-rich fermentation medium. Even though this pellet-like morphology allowed easier mixing and better mass transfer, pellet-like mycelia were highly sensitive to shear stress (Fig. 2b). No agitation combined with 1.2 vvm aeration was used to maintain this pellet-like morphology during the 2-day lag phase. During the fermentation period, the pellet-like particles became fragmented into both small pellets and filamentous mycelia. As the cultivation processed, the pellet-like cores became smaller, and more cell material displaying the pellet-like morphology was formed. After 132 h, the cells started to autolyse, and particles with a rice-shaped morphology were formed (Fig. 2c). This rice-shaped morphology proved to be optimal during the stage at which a high agitation rate was used to maintain the DO level.

### Development of a multi-stage strategy for ARA fermentation

In this paper, the effects of aeration and agitation on cell morphology, lipid accumulation, and ARA production were investigated systemically, and a multi-stage strategy was developed aimed at achieving a high cell density, high accumulation rate, and high ARA yield. The final dry cell weight, total lipids, ARA contents, and ARA yield reached 41.41, 22.17 g/L, 61.05%, and 13.53 g/L, respectively. The highest ARA productivity obtained in this study, which stood at 1.81 g/L day^−1^, was achieved using the stepwise aeration and agitation control developed here. This was the same as the highest value published for *M. alpina* ME-1 (Jin et al. 2008) and was much higher than the 1.50 g/L day^−1^ reported for *M. alpina* DSA-12 using conventional protocols (Hwang et al. 2005).

Compared to the standard batch and fed-batch fermentation protocols, this multi-stage culture method prolongs the fermentation period by nearly 24 h, and while the total consumption glucose also increased sharply from 80 to 100 g/L, an obvious increase of dry cell weight was also noticed (41.4 g/L). ARA productivity consequently increased to 1.81 g/L day^−1^, which was 1.08-fold higher than in the fed-batch fermentation.

To optimally analyze the process of cell growth and ARA accumulation, as well as to understand the effects of aeration and agitation on mycelial morphology, the fermentation process was divided into four stages according to cell growth characteristics (Ren et al. 2010). Stage I represents the beginning of the process until the morphological adaptation period; stage II was the phase of high cell density fermentation; stage III encompasses the lipid biosynthesis period; and stage IV comprises the period of most efficient ARA accumulation.

High cell density is the first precondition for high production of intracellular products, and it was obvious that high aeration was beneficial to cell growth. Our study also showed this positive effect, but with a reduction of *Y*_x/s_ at stage II and a slight increase at stage III (Table 1). This might be explained by the fact that cell respiration would be intensified and additional carbon flux channeled towards the tricarboxylic acid cycle under a high aeration rate. Consequently, dissolved oxygen can be controlled by using an appropriate aeration and agitation rate. To maintain the dissolved oxygen concentration, usually either an oxygen-enrichment method or a pressurization method is used (Higashiyama et al. 2002). In this research, on the other hand, the DO concentration was maintained via a combined stepwise aeration and agitation control strategy.

**Table 1:** Comparison of fermentation parameters at different stages of ARA fermentation via a multi-stage fermentation strategy

	Stage			
	I	II	III	IV
Glucose consumption rate (g/L h^−1^)	0.479 ± 0.28	1.104 ± 0.19	0.694 ± 0.29	None
ARA increase (%)	None	10.676 ± 0.81	2.492 ± 0.13	18.862 ± 0.23
*Y*_x/s_	0.614 ± 0.21	0.373 ± 0.32	0.419 ± 0.18	None
*Y*_l/s_	0.165 ± 0.33	0.208 ± 0.38	0.341 ± 0.21	None
*Y*_ARA/s_	0.048 ± 0.33	0.098 ± 0.33	0.159 ± 0.18	None

*Y*_x/s:_ conversion of glucose to biomass

*Y*_l/s:_ conversion of glucose to lipids

*Y*_ARA/s_: conversion of glucose to ARA

**Fig. 3:**
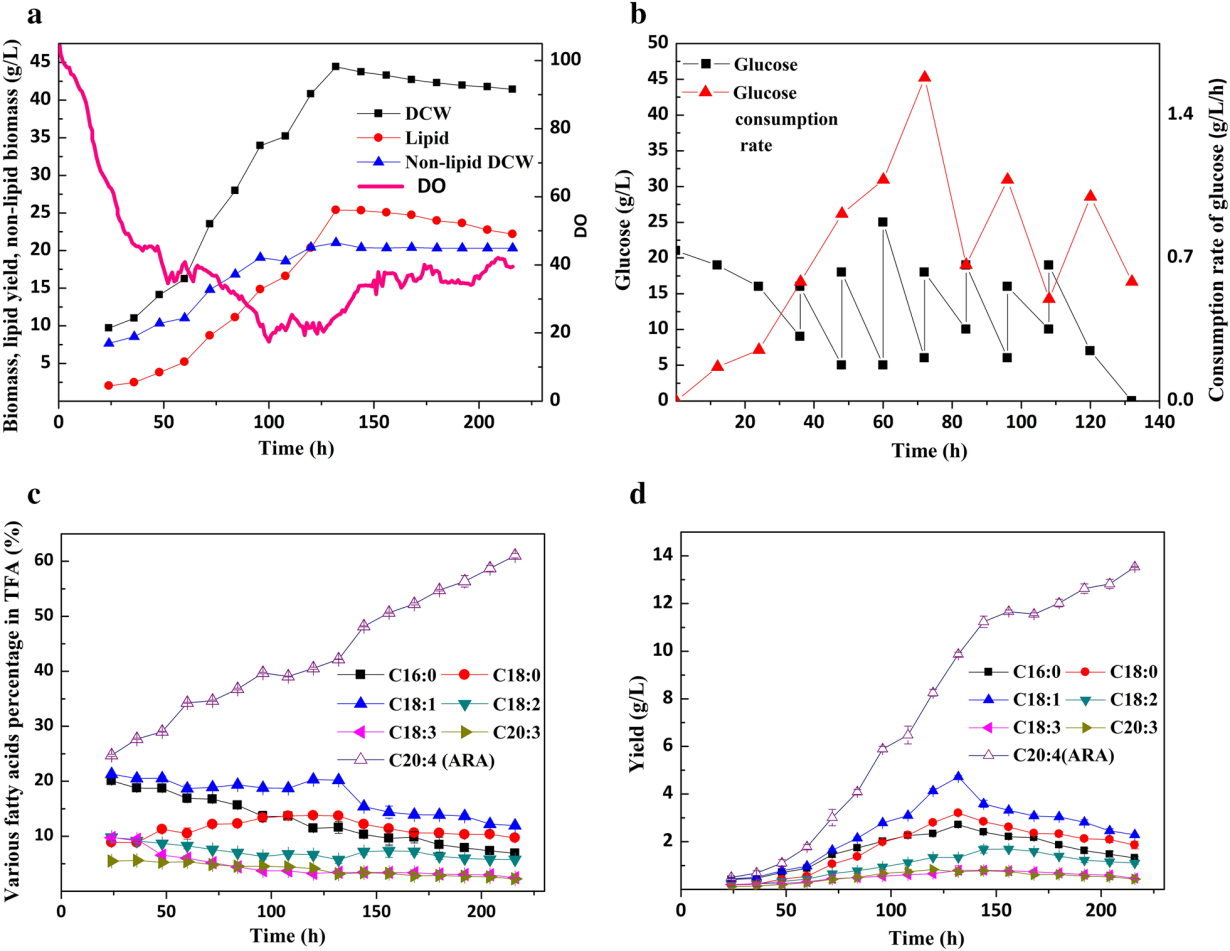
Time-course of various fermentation parameters during the production of high-quality ARA-rich oil via a multi-stage fermentation strategy. **a** Biomass, lipid yield, non-lipid biomass, and DO; **b** glucose and glucose consumption rate; **c** percentage of various fatty acids in TFA; **d** concentration of various fatty acids. The shown values represent the averages ± SD from three independent experiments

During stage I (from 0 to 48 h), glucose was consumed to below 10 g/L after a single feeding. Nitrogen was considered to be exhausted at 48 h (Lu et al. 2011; Ling et al. 2016). With ample carbon and nitrogen source, the dry cell weight increased slightly to 13 g/L, albeit with only 3 g/L of lipids and nearly 10 g/L of non-lipid dry cell weight. At the same time, pellet-like morphology could be preserved better. In stage II (from 48 to 96 h), the agitation rate was increased stepwise from 50 to 150 rpm, while the DO concentration was maintained between 10 and 20%. The glucose consumption rate reached its maximum, which might be explained by increased consumption for cell maintenance. At this stage, the glucose consumption rate was so high that glucose needed to be fed every 12 h, and a sharp increase of biomass from 13 to 28 g/L was also noticed. In stage III (from 96 to 132 h), the dry cell weight reached its maximum value of 44.2 g/L, whereas the non-lipid dry cell weight increased slightly and remained at a constant level. At the same time, the lipid contents increased from 11 to 25 g/L (Fig. 3), which indicated that cell metabolism had shifted away from cell growth towards lipid accumulation. After the seventh batch of glucose feed, the consumption rate began to decline at 96 h. Moreover, the ARA contents slightly decreased, and other fatty acids (such as C18:0 and C18:1) increased, which can likely be explained by the high accumulation of lipid droplets.

High ARA content in total fatty acids is the prerequisite for high-quality ARA-rich oil, and a high aeration rate was favorable for efficient ARA biosynthesis. It is reported that the pathway of ARA biosynthesis was most widespread in oleaginous yeasts, and fungi encompasses both desaturation and elongation steps (Ji et al. 2014a). Palmitic acid (C16:0) is the main saturated fatty acid formed by fatty acid synthase (FAS), and ARA is subsequently produced from it via desaturase and elongase reactions. Our study showed that adequate oxygen was needed to increase the levels of unsaturated fatty acids, especially C18:3 and C20:4 (ARA). In stage IV (from 132 to 216 h), an improved mycelium-aging protocol was used to enhance ARA production (Zhang et al. 2015). When cells were cultivated under high aeration and agitation rates (Fig. 3), glucose was exhausted at 132 h, with dry cell weight reaching 44.2 g/L. Overall, the cells consumed 100 g/L of glucose, which was 20 g/L more than in fed-batch fermentation. The main increase in ARA contents was observed during stage IV, at which point the ARA contents reached 61.1%, with total yield also increasing sharply.

### Changes in PUFA distribution in response to different dissolved oxygen conditions

**Fig. 4:**
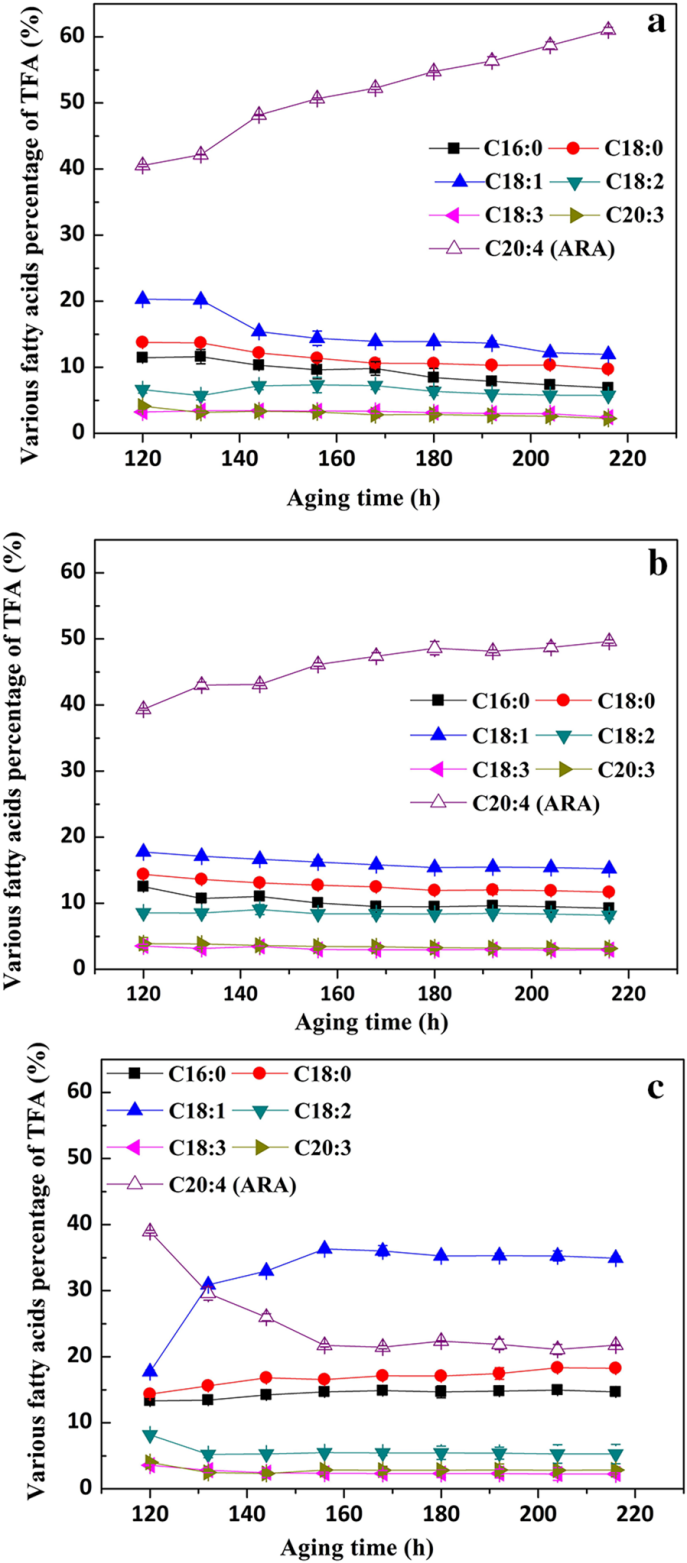
Effect of different DO concentrations on the accumulation of ARA-rich oil during aging. **a** DO kept between 30 and 40%; **b** 10–20%; **c** 0–5%

PUFAs are produced via desaturation and elongation reactions, which involve aerobic oxygenation. Therefore, dissolved oxygen (DO) is a very significant factor for PUFA production, as reported by previous studies (Higashiyama et al. 2002; Su et al. 2016). There have also been some attempts to monitor and control the DO concentration in order to prevent DO limitation during ARA production (Higashiyama et al. 1999; Totani et al. 1992). Cultivations were carried out at different DO levels in the range of 30–40, 10–20, and 0–5%, respectively, and the optimum DO concentration range was found to be 30–40%, as shown in Fig. 4. No significant differences were observed in the contents of C18:2, C18:3, and C20:3 among the 30–40, 10–20, and 0–5% DO groups. The contents of C16:0 and C18:0 were increased slightly at 30–40 and 10–20% DO, respectively, compared to 0–5%. In this optimal DO concentration range, the ARA yield was enhanced about 1.2-fold compared to that obtained at 10–20%, and ARA contents decreased drastically from 39.4 to 22.8% at 0–5% DO. This decrease was likely due to stress caused by the very limited DO concentration. This observation underscores that DO concentration is indeed one of the most important factors influencing ARA productivity.

### Kinetic parameters of the multi-stage strategy

To analyze the kinetic characteristics of the multi-stage fermentation process, five parameters, including glucose consumption rate, ARA increase rate, *Y*_x/s_, *Y*_l/s_, and *Y*_ARA/s_, were compared at different stages. The corresponding data are summarized in Table 1. The values of these kinetic parameters all fluctuated with time and were especially influenced by glucose feeding. At the early fermentation stage (stage I), high aeration without mechanical (Table 1) agitation was able to ensure a higher *Y*_x/s_ (conversion of glucose to biomass) than was observed at the other stages. This indicates that high aeration could not only improve cell growth and glucose consumption, but could also accelerate the conversion of glucose to biomass. What is more, omitting the mechanical agitation was beneficial for maintaining a pellet-like morphology. At stage II, the value of *Y*_x/s_ showed a decrease, whereas the values of ARA and *Y*_ARA/s_ (conversion of glucose to ARA) increased sharply, indicating that a high lipid accumulation rate could be maintained under the high aeration and low agitation conditions found in stage II. After 96 h, in stage III, the value of lipid had increased significantly, indicating that the ARA contents were slightly decreased, whereas the other fatty acids, especially C18:0 and C18:1, increased sharply. After 132 h, the increasing ARA concentration reached the value of 18.9%, while glucose was exhausted. Although the ARA increase at the end of the multi-stage process (18.9%) was higher than during stages I to III, beginning cell autolysis resulted in less glucose consumption, and led to a slight decrease of overall dry cell weight. This further confirmed the importance of the multi-stage strategy combined with an efficient control of mycelial morphology.

To sum up, this study systematically examined the effects of aeration and agitation rates on ARA production by *M. alpina* and proposed a stepwise aeration and agitation rate control strategy to achieve a high cell growth rate and optimal overall productivity (Table 2).

**Table 2:** Comparison of parameters from different ARA fermentation strategies

	Fermentation strategy			Rate of increase^a^
	Batch	Fed-batch	Multi-stage	
DCW (g/L)	31.06	36.08	41.41	14.77%
Mycelial specific growth rate (g/L d^−1^)	4.78	6.01	5.92	−1.50%
Lipids (g/L)	17.63	17.09	22.17	25.75%
Fermentation duration (d)	6.5	6	7.5	7.69%
Total glucose (g/L)	80	80	100	25%
Glucose consumption rate (g/L h^−1^)	0.51	0.56	0.76	35.71%
ARA contents (%)	36.30	42.81	61.05	42.61%
ARA yield (g/L)	6.40	10.01	13.53	35.16%
ARA productivity (g/L d^−1^)	0.98	1.67	1.81	7.78

^a^This value represents the corresponding data from the multi-stage strategy divided by the highest respective value from either the batch or fed-batch fermentation

## Conclusions

This paper compared the experimental results of fermentations using *M. alpina* to produce oils rich in ARA via different culture strategies. The morphology of the fungal mycelia could be maintained in an optimal state throughout the fermentation. It could be shown that the multi-stage strategy provides a favorable gas–liquid mixture, and consequently increase biomass accumulation significantly. This work offers insights into the control of aeration and agitation and provides a reference for the fermentation of filamentous fungi at a mass industrial scale.
